# CD4^+^ T cells reverse surface antigen persistence in a mouse model of HBV replication

**DOI:** 10.1128/spectrum.03447-23

**Published:** 2023-11-10

**Authors:** Jacob T. Bailey, Safiehkhatoon Moshkani, Catherine Rexhouse, Jesse L. Cimino, Michael D. Robek

**Affiliations:** 1 Department of Immunology & Microbial Disease, Albany Medical College, Albany, New York, USA; Kumamoto Daigaku, Kumamoto, Japan

**Keywords:** hepatitis B virus, antibody function, T lymphocytes, B lymphocytes

## Abstract

Hepatitis B virus (HBV) is responsible for over 800,000 deaths yearly worldwide. While chronic HBV infection currently has no highly effective cure, immunotherapy has generated interest as a potential therapeutic solution due to the connection between viral chronicity and the magnitude of HBV-specific immune responses. Suitable animal models for studying successful immune responses to HBV are limited due to the lack of natural viral tropism for rodents. Using a mouse model of HBV replication, we found that CD4^+^ T cells are essential for the antibody-mediated clearance of hepatitis B surface antigen (HBsAg) and that early transient inhibition of the CD4^+^ T cell-B cell axis imparts HBsAg persistence through CD4^+^ T cell functional impairment. HBsAg resolution in BALB/c mice transduced with an adeno-associated virus encoding the HBV genome (AAV-HBV) depended on B cells, MHCII, and CD4^+^ T cells, but not CD8^+^ T cells, indicative of antibody-mediated control. Furthermore, disrupting the CD40-CD40L interaction blunted HBsAg clearance and the generation of HBsAg-specific B cells. Finally, HBsAg persistence induced by transient CD4 depletion was reversible by the transfer of total splenocytes or purified CD4^+^ T cells from immunized mice. These findings demonstrate the T cell-dependent nature of HBsAg-resolving antibody responses as well as the ability of CD4^+^ T cells to reverse HBsAg persistence.

**IMPORTANCE:**

Hepatitis B virus (HBV) is a leading causative agent of viral hepatitis. A preventative vaccine has existed for decades, but only limited treatment options are available for people living with chronic HBV. Animal models for studying HBV are constrained due to narrow viral tropism, impeding understanding of the natural immune response to the virus. Here, using a vector to overcome the narrow host range and establish HBV replication in mice, we identified the role of helper T cells in controlling HBV. We show that helper T cells promote the B cell’s ability to generate antibodies that remove HBV and its associated surface antigen from the blood and that transfer of purified helper T cells from HBV-immunized mice can reverse the accumulation of virus and antigen, furthering our understanding of the immune response to HBV.

## INTRODUCTION

Hepatitis B virus (HBV) is a member of the Hepadnaviridae family, which is characterized by noncytolytic, enveloped, liver-tropic DNA viruses. Despite the availability of an effective preventative vaccine, HBV remains a global health problem, as almost one million people succumb yearly from HBV-related complications, including liver cirrhosis and hepatocellular carcinoma. HBV persistence within infected hepatocytes is primarily mediated by the stability of the HBV transcriptional template known as covalently closed circular DNA (cccDNA) (1, 2). Current antiviral therapies consist of interferon (IFN)-α (3) and nucleos(t)ide analogs (4), which effectively inhibit HBV replication throughout the course of treatment but are limited by their inability to efficiently achieve functional cure (5).

Acute HBV infection is resolved following a robust and multi-specific CD8^+^ T cell response that downregulates HBV replication through antiviral cytokines IFN-γ and TNF-α and eliminates HBV-infected hepatocytes by inducing apoptosis through Fas/FasL and perforin/granzyme B (6 – 9). Less understood is the role of CD4^+^ T cells in HBV control. In viral infections, CD4^+^ helper T cells promote functional antibody responses against T-dependent antigens and support antigen-specific CD8^+^ T cell responses. Helper T cells interact with B cells directly through CD40/CD40L and TCR/MHCII to promote germinal center formation, antibody class switching, and B cell survival (10, 11). Similarly, CD4^+^ T cells interact with conventional type 1 dendritic cells (cDC1) by ligating CD40 on cDC1s to stimulate their maturation and antigen cross-presentation, which, in turn, promotes virus-specific CD8^+^ T cell responses (12, 13). Studies using HBV-infected chimpanzees found that CD4^+^ T cells support cytotoxic lymphocyte (CTL) responses that result in viral clearance. CD4^+^ T cell depletion at the time of infection, but not at later stages, led to viral persistence (6, 14), indicating that CD4^+^ T cells function to prime HBV-specific immune responses.

Despite their lack of natural susceptibility, rodent models of HBV infection are highly desired due to their ubiquity in the laboratory and relative ease of use. In humans, hepatocytes are permissive to HBV infection through virus interaction with the Na^+^-taurocholate co-transporting polypeptide (NTCP) (15). However, murine NTCP is insufficient for HBV entry and, thus, requires an alternative solution to establish replication in mice. Transgenic mice expressing human NTCP allow HBV entry into hepatocytes but lack the cellular machinery needed for efficient cccDNA formation, restricting replication (16). HBV transgenic mice exhibit antigen persistence but are tolerized, resulting in the inactivation and deletion of HBV-specific lymphocytes (17), thus complicating the investigation of some HBV-specific immune responses. A more recent model of murine HBV infection utilizes AAV-HBV to establish persistent replication in immunocompetent mice (18 – 20). An advantage of the AAV-HBV model compared to HBV transgenic mice is that it permits the study of early virus-host interactions and immunological events that occur following the initial encounter by the immune system of HBV antigens produced in the liver, thus mimicking the very early stages of HBV infection (18 – 20).

The AAV-HBV model of HBV infection using C57BL/6 mice induces viral persistence characterized by stable HBsAg and HBeAg expression, which is believed to contribute to immune dysfunction and the inability to resolve infection spontaneously (18, 21). However, the absence of a measurable cellular or humoral response without immunization or experimental immune activation proves challenging for evaluating natural immunological events leading to HBV resolution. Here, we report the antibody-mediated nature of HBsAg resolution in BALB/c mice transduced with AAV-HBV. Furthermore, this response exhibits classical features of T cell help, necessitating antigen-dependent CD4^+^ T cells and their associated signaling pathways at the time of transduction for functional HBsAg clearance. Finally, HBsAg persistence following transient CD4 depletion was reversed by transferring total splenocytes or purified CD4^+^ T cells from HBsAg-immunized mice. Together, our findings further define the role of CD4^+^ T cell help for spontaneous anti-HBsAg antibody (HBsAb) generation and highlight the long-term immune impairment resulting from their dysregulation during initial antigen encounter.

## RESULTS

### HBsAg is spontaneously resolved in AAV-HBV mice by HBsAb

**Fig 1:**
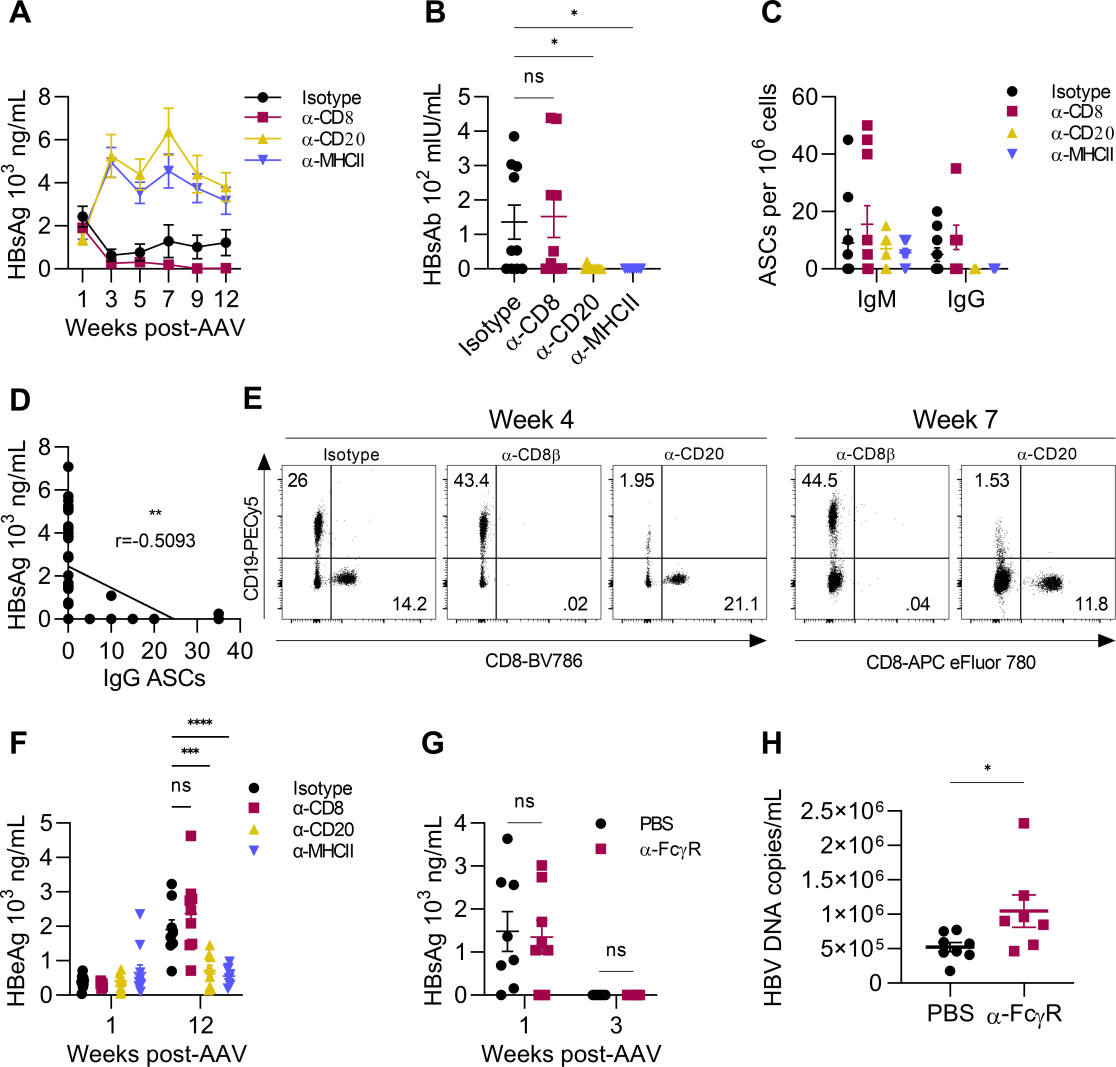
HBsAg is spontaneously resolved in AAV-HBV mice by HBsAb. AAV-HBV-transduced BALB/c mice received 10 doses of depleting (α-CD8, α-CD20), blocking (α-MHCII), or control antibody injections beginning before transduction and ceasing at week 4 post-transduction. (**A**) Serum HBsAg was measured over time by ELISA. (**B**) Serum HBsAb was measured at week 13 by ELISA. (**C**) Splenic IgM and IgG antibody-secreting cells (ASCs) specific for HBsAg were measured by dual-color ELISPOT at the experiment endpoint (week 13). (**D**) Correlation of HBsAg vs anti-HBsAg IgG-producing ASCs. (**E**) Representative flow cytometry plots of lymphocytes in blood at 4 and 7 weeks following depletion of CD8^+^ or CD20^+^ cells (gated on single cells/CD45^+^). (**F**) Serum HBeAg was measured at weeks 1 and 12 by ELISA. (**G**) Serum HBsAg was measured by ELISA at weeks 1 and 3 post-transduction in mice receiving α-FcγR. (**H**) Serum HBV DNA was determined by qPCR at week 3 in mice administered α-FcγR. *n* = 8–10 mice per group. *P < 0.05, **P < 0.01. Mann-Whitney test, one- or two-way ANOVA, or Spearman correlation was used to determine statistical significance.

C57BL/6 and HLA-A2/HLA-DR1 transgenic mice transduced with AAV-HBV exhibit persistent HBsAg and HBeAg expression (18, 19). First-generation offspring from C57BL/6 crossed with BALB/c (CB6F1; H-2^bxd^) mice generate anti-HBsAg antibody responses following AAV-HBV transduction (22). Similarly, BALB/c mice hydrodynamically transfected with an HBV plasmid undergo antibody seroconversion and clear HBsAg though other unknown immunological divergences further drive clearance of HBV DNA and HBeAg in this model (23). To understand which immunological components are required for HBsAg clearance in AAV-HBV transduced BALB/c mice, CD8^+^ T cells or B cells were depleted beginning 1 week before AAV-HBV transduction and ending 4 weeks post-transduction. Additionally, to evaluate the role of CD4^+^ T cell help without depleting CD4^+^ dendritic cells, major histocompatibility complex II (MHCII) was blocked with α-MHCII antibody. CD8^+^ T cells are well-defined contributors to HBV defense in humans and non-human primates (7, 9, 24, 25), and the magnitude of their response is a significant determinant of disease outcome (25). CD8^+^ cell depletion in AAV-HBV mice did not impair HBsAg resolution, suggesting that spontaneous HBsAg clearance is CD8^+^ T cell-independent (Fig. 1A). In contrast, B cell-depleted and MHCII-blocked AAV-HBV mice failed to restrain HBsAg (Fig. 1A), indicating control is both antibody-mediated and antigen-dependent. Serum HBsAb was detectable in isotype-control (rat IgG2b; matched to α-MHCII) and CD8-depleted mice but not in B cell-depleted or MHCII-blocked mice (Fig. 1B). Furthermore, B cells producing HBsAg-specific IgG were only detectable in control and CD8-depleted but not B cell-depleted or MHCII-blocked mice (Fig. 1C). HBsAg and HBsAg-specific IgG-producing B cells were negatively correlated as determined by Pearson correlation (*r* = −0.5093; *P* = 0.0013) (Fig. 1D), suggesting that IgG is essential for HBsAg control. CD8^+^ T cell and B cell depletion was confirmed at weeks 4 and 7 post-transduction (Fig. 1E). HBeAg increased over time in the serum of control and CD8-depleted mice but, unexpectedly, was reduced in CD20-depleted and MHCII-blocked mice (Fig. 1F). To understand the role of the Fc receptor in HBsAg resolution, FcγR (CD16/CD32) was inhibited by administering blocking antibody beginning before AAV-HBV transduction. HBsAg became undetectable similar to PBS control mice by week 3 post-transduction (Fig. 1G). However, HBV DNA in the serum of FcγR-blocked animals increased by an average of one log (Fig. 1H), indicating an Fc-dependent mechanism of virion reduction. These results demonstrate the antibody-dependent manner by which BALB/c AAV-HBV mice spontaneously resolve HBsAg.

### Transient CD4^+^ T cell depletion induces HBsAg persistence

**Fig 2:**
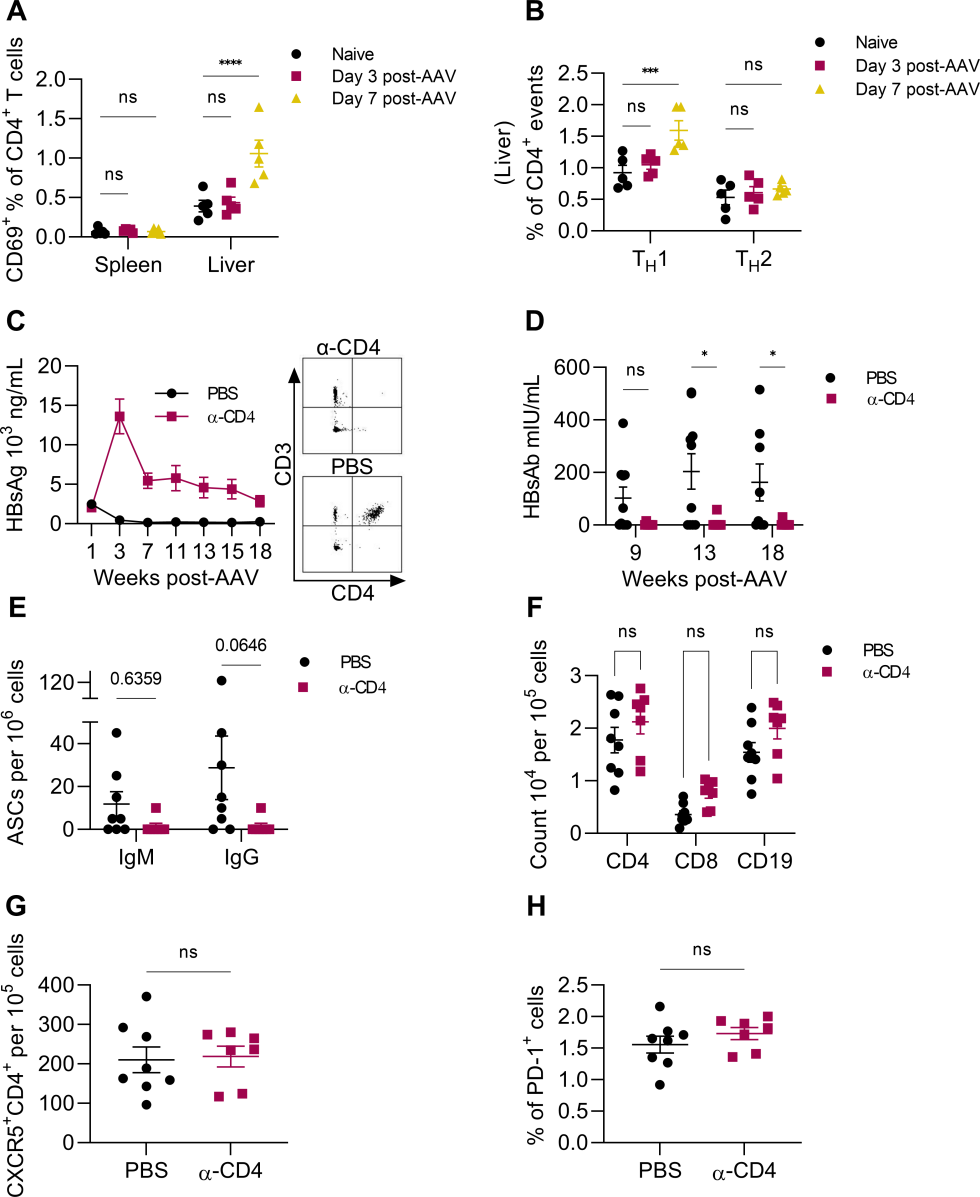
Transient CD4^+^ T cell depletion induces HBsAg persistence. CD4^+^ T cell activation was evaluated following AAV-HBV transduction. (**A**) CD69 and (**B**) intracellular IFN-γ (T_H_1) and IL-4 (T_H_2) were measured by flow cytometry at 3 and 7 days post-AAV-HBV transduction. Populations in (**A**) were gated on single cells/lymphocyte size exclusion/CD4^+^/CD69^+^ and populations in (**B**) were gated on single cells/lymphocyte size exclusion/CD4^+^/IFN-γ^+^ or IL-4^+^; *n* = 5. AAV-HBV mice received 10 doses of α-CD4 or vehicle (PBS) beginning before transduction and continuing until cessation at week 4. Serum (**C**) HBsAg and (**D**) HBsAb were measured over time by ELISA in AAV-HBV transduced mice depleted of CD4^+^ T cells. (C, right) CD4^+^ T cell depletion determined by flow cytometry from whole blood at week 7. (**E**) Splenic IgM and IgG antibody-secreting cells (ASCs) specific for HBsAg were measured by dual-color ELISPOT at the experimental endpoint (week 18). (**F, G**) Lymphocyte count from spleen (week 18) as determined by flow cytometry. CD4, CD8, and CD19 were gated on single cells and lymphocyte size exclusion. (**G**) CXCR5^+^CD4^+^ event count determined by flow cytometry. Population gated on single cells and lymphocyte size exclusion. (**H**) Percent PD-1^+^ splenocytes were gated on single cells and lymphocyte size exclusion. *n* = 7–10 mice per group. A–B and C–H are separate experiments. *P < 0.05, ***P < 0.001, ****P < 0.0001. Statistical significance was determined by Student’s *t*-test or two-way ANOVA.

CD4^+^ T cells are an integral component of antibody maturation, providing help to B cells through direct (receptor/ligand) and indirect (cytokine) interactions. CD4^+^ T cell help during an immune response, among other functions, promotes germinal center formation in addition to antibody class switching, promoting a more robust antibody response. CD4^+^ T cell activation was measured by CD69 and cytokine expression in the spleen and liver following AAV-HBV transduction. By day 7 post-transduction, CD69 expression (Fig. 2A) and IFN-γ expression by liver CD4^+^ T cells (Fig. 2B) were elevated compared to naïve mice, indicating activation of CD4^+^ T cells following transduction. To understand the contribution of CD4^+^ T cells in HBsAg clearance, CD4^+^ T cells were depleted before AAV-HBV transduction. CD4^+^ T cell depletion was confirmed at week 7 post-transduction (Fig. 2C, right). Antigenemia was increased in CD4-depleted mice at week 3 post-transduction and remained elevated over 18 weeks (Fig. 2C, left). Serum HBsAb was mostly undetectable in CD4-depleted mice (Fig. 2D), providing evidence that CD4^+^ T cells are necessary for a functional anti-HBsAg antibody response. HBsAg-specific IgG-producing B cells were reduced in CD4-depleted mice at week 18 (Fig. 2E). Since α-CD4 treatment is transient and ceased at week 4 post-transduction, spleens were harvested to determine the impact of CD4 depletion on lymphocyte populations. Although CD4^+^ and CD8^+^ T cell and CD19^+^ B cell populations were comparable in CD4-depleted and control (PBS) mice at 14 weeks following cessation of antibody administration (Fig. 2F), HBsAg clearance and a measurable HBsAb response were restricted to control mice (Fig. 2C and D). T follicular helper (T_FH_) cells, defined by CD4 and CXCR5 expression (26), were measured to determine if the inability to mount an anti-HBsAg antibody response resulted from prolonged T_FH_ cell reduction. T_FH_ cells were similar in frequency in CD4-depleted compared to control mice (Fig. 2G). As measured by PD-1 expression, lymphocyte exhaustion was also not significantly increased in splenocytes of CD4-depleted mice at week 18 (Fig. 2H). Together, these findings demonstrate the necessity of CD4^+^ T cells for spontaneous HBsAg resolution and suggest that an absence of T cell help during initial antigen encounter promotes irreversible HBsAg persistence, regardless of subsequent CD4^+^ T cell repopulation.

### The anti-HBsAg antibody response is dependent on the CD40/CD40L axis

**Fig 3:**
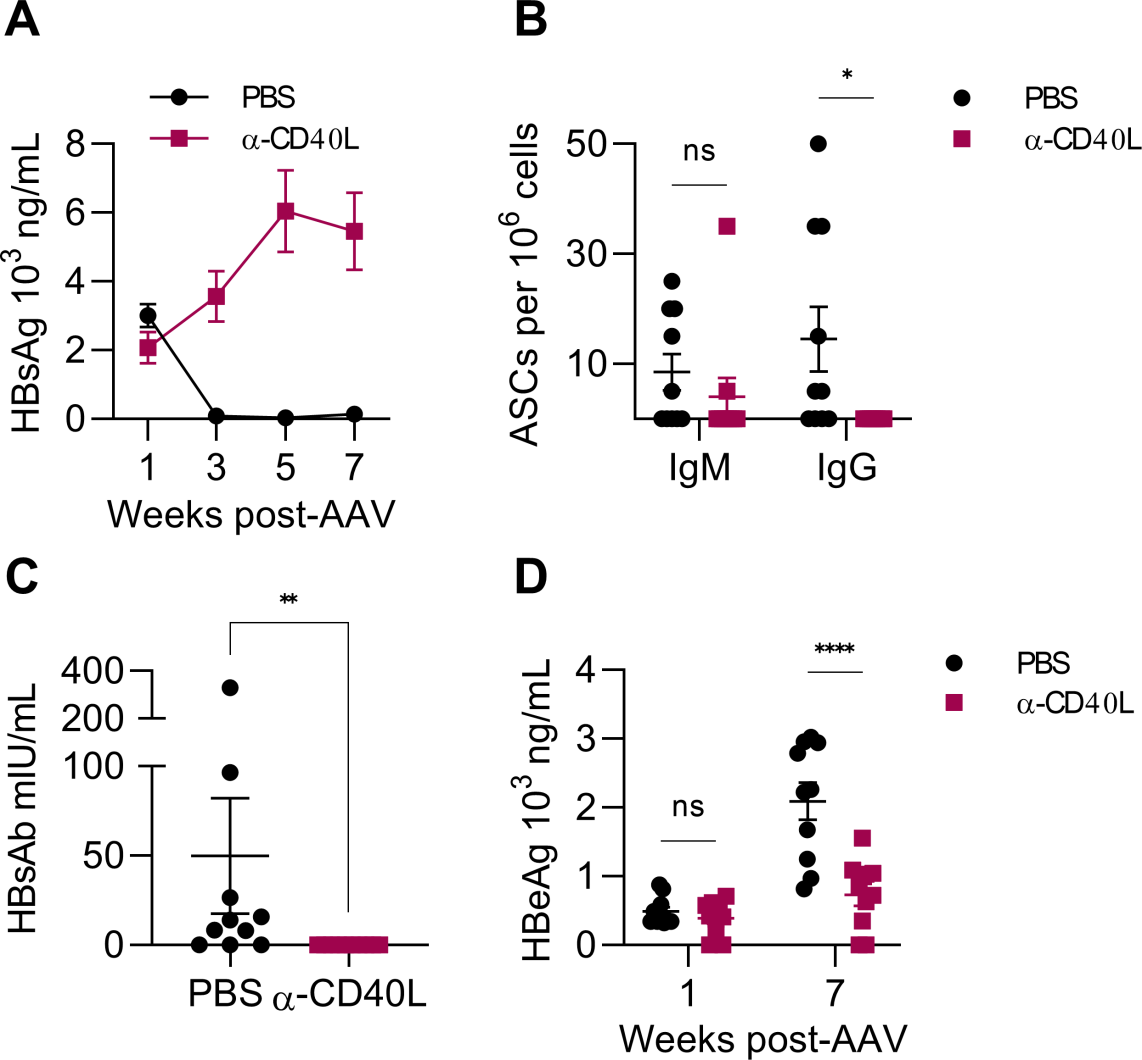
HBsAg clearance in BALB/c mice depends on the CD40/CD40L axis. AAV-HBV mice received 10 doses of CD40L-blocking antibody (α-CD40L) or vehicle (PBS) beginning before transduction and continuing until cessation at week 4. (**A**) Serum HBsAg was measured biweekly in CD40L-blocked AAV-HBV mice by ELISA. (**B**) Splenic IgM and IgG antibody-secreting cells (ASCs) specific for HBsAg were measured by dual-color ELISPOT at the experimental endpoint (week 7). (**C**) Serum HBsAb was measured by ELISA at week 7. (**D**) Serum HBeAg was measured by ELISA at weeks 1 and 7 post-AAV-HBV transduction. All graphs are from the same experiment. *n* = 10 mice per group. *P < 0.05, **P < 0.01, ****P < 0.0001. Statistical significance was determined by the Mann-Whitney test or two-way ANOVA.

Since HBsAg is a T-dependent antigen, we next examined the necessity of CD40 signaling for the anti-HBsAg antibody response. CD40 is a costimulatory molecule expressed primarily by B cells and antigen-presenting cells (27). As a member of the TNF-α receptor superfamily, its ligation results in downstream signaling that supports cell activation and survival (28). In the context of T cell help, CD40L, primarily expressed by CD4^+^ T cells, ligates CD40 on B cells to promote germinal center formation, leading to class-switching and affinity maturation of antibody responses (29). To understand the role of CD40 signaling in forming the anti-HBsAg antibody response, CD40L was blocked with an inhibitory antibody before AAV-HBV transduction. CD40L-blocked mice failed to clear HBsAg over the 7-week duration of the study (Fig. 3A). Because spontaneous HBsAg clearance in AAV-HBV-transduced BALB/c mice is HBsAb-dependent, we next examined components of the humoral immune response. IgG-producing HBsAg-specific B cells isolated from the spleen were decreased at week 7 in CD40L-blocked mice (Fig. 3B), suggesting impaired class switching capacity. Similarly, antibody specific for HBsAg was undetectable in CD40L-blocked mice at week 7 (Fig. 3C), indicating that CD40/CD40L interactions are required to generate a functional anti-HBsAg antibody response. Interestingly, while unlikely related to antibody, serum HBeAg was reduced in CD40L-blocked mice at week 7 post-transduction (Fig. 3D). This effect was also observed in MHCII-blocked and CD20-depleted mice (Fig. 1F) and suggests that a population of CD4^+^ T cells may influence HBV gene expression through CD40L, presumably as a consequence of interactions with CD40 on antigen-presenting cells.

### Splenocyte transfer resolves HBsAg persistence in AAV-HBV mice

**Fig 4:**
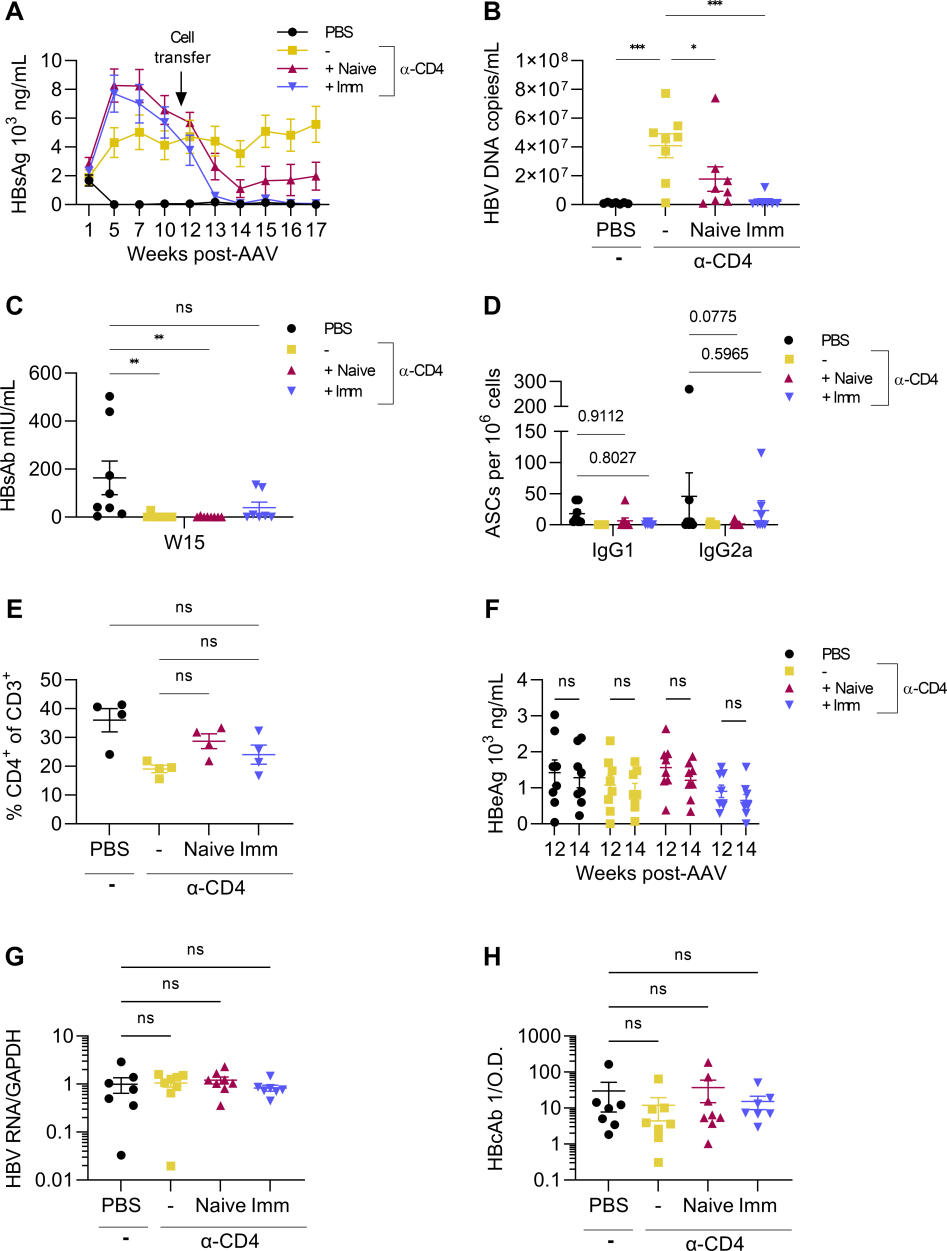
Total splenocyte transfer reverses HBsAg persistence in AAV-HBV mice. HBsAg persistence was induced in AAV-HBV-transduced mice by transient CD4 depletion, beginning before transduction and ending on week 4 for a total of 10 doses of CD4-depleting antibody. Following the repopulation of CD4^+^ T cells, splenocytes from naïve or immunized mice were transferred into HBsAg-persistent recipients (week 11). (**A**) Serum HBsAg was measured by ELISA over time in AAV-HBV transduced mice. (**B**) HBV DNA was isolated from serum at week 17 post-transduction and measured by qPCR. (**C**) Serum HBsAb was measured at week 15 by ELISA. (**D**) Splenic IgG1 and IgG2a antibody-secreting cells (ASCs) specific for HBsAg were measured by dual-color ELISPOT at week 18. (**E**) CD4^+^ T cell count from blood (week 16) as determined by flow cytometry. CD3 and CD4 were gated on single cells and lymphocyte size exclusion. *n* = 4. (**F**) HBeAg was measured in serum at weeks 12 and 14 post-transduction. (**G**) Liver HBV gene expression was measured at week 17 by RT-qPCR. (**H**) Serum HBcAb as determined by ELISA at week 17. Groups shown are PBS, no splenocyte transfer (–), naïve splenocyte recipients (naïve), and immunized splenocyte recipients (Imm). All graphs are from the same experiment. *n* = 6–8 mice per group. *P < 0.05. Statistical significance was determined by Student’s *t*-test, one-way ANOVA, or two-way ANOVA.

We observed that long-term numerical lymphocyte differences are not responsible for the loss of extended HBsAg clearance following CD4 depletion. Thus, we sought to determine whether the loss of HBsAg resolution following CD4 depletion is due to functional discrepancies in repopulating splenocytes. To determine if immune dysfunction arising from transient CD4^+^ T cell depletion is reversible, naïve or HBV-immunized splenocytes were transferred into HBsAg-persistent AAV-HBV mice following CD4^+^ T cell repopulation. Recipients of both naïve and HBV-immunized splenocytes experienced significant HBsAg reduction by week 2 post-transfer (week 13). However, as expected, recipients of HBV-immunized splenocytes controlled HBsAg more effectively (Fig. 4A). Likewise, serum HBV DNA was reduced in recipients of naïve and immunized splenocytes at week 6 post-transfer (week 17), with the latter offering the greatest reduction in peripheral virions (Fig. 4B). At week 4 post-transfer (week 15), HBsAb was detectable in most PBS control mice but essentially undetectable in either naïve or HBV-immunized splenocyte recipients (Fig. 4C). Serum antibody measurements are indicative of a high antibody/antigen ratio since immune complexes can be difficult to detect by antibody ELISA (30). HBsAg-specific IgG1 and IgG2a B cells obtained from the spleen were measured at the experimental endpoint (week 17). HBsAg-specific IgG2a B cells were comparable between control mice and HBV-immunized splenocyte recipients though naïve splenocyte recipients remained mostly undetectable (Fig. 4D). At week 16, CD4^+^ T cell frequencies were similar in groups receiving transient CD4 depletion (Fig. 4E), absolving numerical differences in CD4^+^ T cells as drivers of HBsAg clearance following cell transfer. HBeAg was unaffected by splenocyte transfer (Fig. 4F), exonerating virus-specific CD8^+^ T cells as the drivers of HBsAg clearance following splenocyte transfer. To confirm that HBsAg control is not a consequence of altered HBV gene expression, HBV RNA was measured at week 17. HBV gene expression was unaffected by cell transfer as determined by HBV RNA levels (Fig. 4G). Hepatitis B core antibody (HBcAb) was also measured in the serum at week 17 though no changes were observed between groups (Fig. 4H). These results imply that naïve or HBV-immunized splenocyte transfer is sufficient for reversing immune dysfunction arising from transient CD4 depletion.

**Fig 5:**
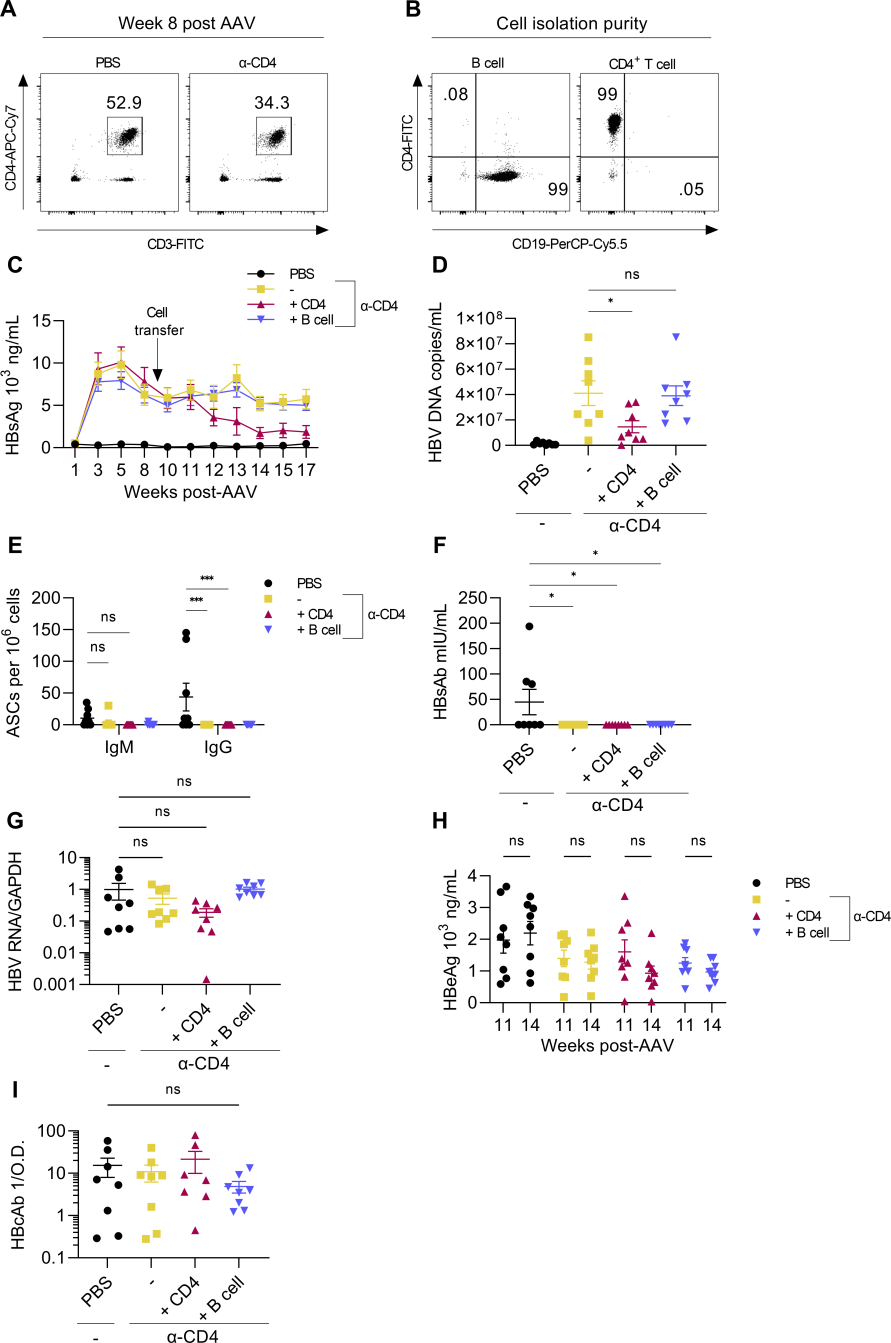
CD4^+^ T cell transfer from HBsAg-immunized mice reverses HBsAg persistence in AAV-HBV mice. HBsAg-persistent AAV-HBV mice generated by transient CD4 depletion (two doses of α-CD4) beginning before transduction received purified CD4^+^ T cells or B cells from HBsAg-immunized mice at week 9 post-transduction. (**A**) CD4^+^ T cell repopulation before cell transfer was measured by flow cytometry at week 8 post-AAV-HBV transduction. (**B**) B cell (left) and CD4^+^ T cell (right) purity following magnetic column separation was determined by flow cytometry. (**C**) Serum HBsAg was measured over time by ELISA. (**D**) HBV DNA from serum was measured at week 17 post-transduction by qPCR. (**E**) Splenic IgM and IgG antibody-secreting cells (ASCs) specific for HBsAg were measured by dual-color ELISPOT at week 17. (**F**) HBsAb was measured in serum at week 17 post-transduction by ELISA. (**G**) Liver HBV gene expression was determined by RT-qPCR at week 17. (**H**) Serum HBeAg was measured by ELISA at weeks 11 and 14 post-transduction. (**I**) Serum HBcAb was measured by ELISA at week 17. All graphs are from the same experiment. *n* = 8 mice per group *P < 0.05, ***P < 0.001. Statistical significance was determined by Student’s *t*-test, one-way ANOVA, or two-way ANOVA.

Since total splenocyte transfer restored HBsAg clearance, we next sought to determine whether persistence resulted from specifically CD4^+^ T cell or B cell dysfunction by transferring purified CD4^+^ T cells or B cells from HBsAg-immunized mice. BALB/c mice received 2 doses (instead of 10 as in Fig. 4) of CD4-depleting antibody before AAV-HBV transduction to induce HBsAg persistence. CD4^+^ T cells were measured at week 8 to confirm their repopulation prior to the transfer of either CD4^+^ T cells or B cells from immunized mice (Fig. 5A). Donor mice were immunized and boosted as in Fig. 4. At week 9 post-AAV-HBV transduction, splenocytes from immunized donor mice were isolated and CD4^+^ T cells and B cells were purified for transfer. Fractions of CD4^+^ T cells and B cells were both 99% pure as determined by flow cytometry (Fig. 5B). Purified CD4^+^ T cells or B cells from immunized mice were then transferred to HBsAg-persistent mice, and serum HBsAg was measured weekly. Serum HBsAg declined in CD4^+^ T cell recipients beginning 3 weeks post-transfer (week 12) and plateaued at 5 weeks post-transfer (Fig. 5C). HBV DNA in serum mirrored HBsAg in that CD4^+^ T cell transfer reduced detectable virions compared to mice that did not receive CD4^+^ T cells or B cells (Fig. 5D). B cell recipients could not control HBsAg or virions in any capacity, indicating that HBsAg persistence results primarily from the dysfunction of CD4^+^ T cells. Serum HBsAb (Fig. 5F) and splenic α-HBsAg IgG-producing B cells (Fig. 5E) were detectable in PBS control mice but undetectable in CD4^+^ T cell and B cell recipients at week 8 post-transfer (week 17). The absence of detectable serum HBsAb in CD4^+^ T cell recipient mice is likely caused by the presence of HBsAg/HBsAb immune complexes that the ELISA cannot detect. To confirm that HBsAg control following CD4^+^ T cell transfer is not a result of altered HBV gene expression, intrahepatic HBV RNA and serum HBeAg were measured. HBV RNA was comparable between groups at week 17 (Fig. 5G), and HBeAg was unchanged between weeks 11 and 14 post-transduction (Fig. 5H). HBcAb was detectable in the serum of mice at week 17 though no differences were observed between groups (Fig. 5I). These findings further support HBsAb production as the mechanism by which CD4^+^ T cell transfer reverses HBsAg persistence. Thus, CD4^+^ T cells from immunized mice can restore HBsAg clearance when transferred to antigenically persistent mice.

## DISCUSSION

Here, we describe the role of CD4^+^ T cells in promoting HBsAg seroconversion by providing T cell help to B cells. We show that the classical model of T cell help applies in that CD4^+^ T cells are required for HBV-specific humoral immunity at the initial moments of antigen encounter and that HBsAg persistence resulting from transient CD4 depletion can be restored by total splenocytes or CD4^+^ T cells but not B cells alone. Our study also emphasizes the differential immune responses to AAV vectors based on the genetic background of the mice. Such differences have also been described using non-HBV AAV vectors that induce T cell tolerance in C57BL/6 but not BALB/c mice (31).

In the context of AAV-HBV, cellular immune tolerance was observed independent of genetic background, while humoral tolerance is observed only in C57BL/6 mice. This same phenomenon was observed in the hydrodynamic model of HBV infection via transfection with pAAV-HBV in which BALB/c mice developed detectable HBsAb by day 14 while C57BL/6 mice remained undetectable throughout day 28 post-transfection (23). In our study, mice were transduced with a specific dose of AAV-HBV to generate approximately 2 µg/mL of serum HBsAg at peak antigenemia. At this dose, HBsAb causes HBsAg to become undetectable in most mice over time. However, some mice did not have detectable levels of HBsAb, indicating an equilibrium of antibody-antigen in the blood. Therefore, it remains possible that higher levels of HBV antigenemia may not be as efficiently controlled by antibody in BALB/c mice.

Immune tolerance is a hallmark feature of chronic HBV and is recapitulated to varying degrees in AAV-HBV mice. While some specific features of both acute and chronic HBV (i.e., detectable liver inflammation, HBV-specific CD8^+^ T cells) are absent in AAV-HBV mice, this model is particularly useful for evaluating immune tolerance as well as spontaneous HBsAg seroconversion in BALB/c mice. HBV transgenic mice are another popular model for investigating immune responses to HBV, though with limitations. In this model, HBV is expressed during early developmental stages, and the immune system exhibits signs of central and peripheral tolerance (32). Additionally, only a portion of transgenic mice spontaneously produce HBsAb, which complicates the investigation of the natural immunological events leading to HBsAg loss. Conversely, AAV-HBV transduced BALB/c mice consistently undergo HBsAg seroconversion within 3 weeks of HBV establishment. Regardless of the chosen model, HBsAb production does not result in the clearance of HBV from the mouse liver. While seroconversion in acutely infected non-human primates is associated with liver inflammation and loss of detectable intrahepatic HBV gene expression (6), a functional cure for chronic HBV may be achieved by HBsAb and loss of detectable blood HBsAg and virions. As such, the AAV-HBV model is well suited for investigating HBsAg seroconversion.

T cell help is an essential component of antibody maturation for T-dependent antigens. Some known consequences of T cell help include promoting survival, proliferation, differentiation, and antibody maturation that ultimately result in a functional and robust humoral response (11). However, chronic infections, including HBV, downregulate effector responses through repeated antigen encounter leading to reduced function, anergy, and eventually deletion of antigen-specific lymphocytes through a process known as immune tolerance (33, 34). This process might be related to HBsAg persistence following disruption of the CD4^+^ T cell-B cell axis. Following cessation of their experimental depletion, repopulating CD4^+^ T cells return to a high antigen environment which could result in either anergy or deletion of HBsAg-specific helper T cells. Chronic lymphocytic choriomeningitis virus studies have shown that CD4^+^ T cell depletion or impairment at initial antigen encounter precludes antigen-specific antibody (35) and CTL responses (36). HBsAg persistence was reversible via transfer of immunized CD4^+^ T cells, suggesting functional impairment of the host’s repopulating CD4^+^ T cells following transient depletion. While we could not demonstrate the contribution of antigen-experienced versus naïve CD4^+^ T cells, both may contribute to HBsAg clearance as transferred total splenocytes, naïve or immunized, were able to reverse HBsAg persistence.

Vaccine studies have generated interest in preventing and overcoming HBV persistence. Immune tolerance can be prevented by prophylactic immunization with vectors expressing HBV antigens (37), and some can even reduce antigenemia when administered therapeutically (38). The goal of vaccination is ideally to promote virus-neutralizing antibody and multi-specific CD8^+^ T cell responses that prevent reinfection of the liver and eliminate HBV-infected hepatocytes, respectively. Although a preventative HBV vaccine has existed for decades (39), an effective therapeutic vaccine has yet to be approved for clinical application. Therapeutic intervention is critical since vertical transmission of HBV from mother to child often results in a lifelong chronic HBV infection with only suboptimal antiviral treatments available (40). CD4^+^ T cells are an essential regulatory component of HBV-resolving immune responses and should be considered when designing therapeutic vaccines. Others have recently observed that CD4^+^ T cells are required to generate HBsAb following therapeutic immunization in the AAV-HBV model (38). Our findings support this observation and demonstrate the ability of the CD4^+^ T cell to promote antibody responses immediately following antigen encounter. Moreover, HBsAg seroconversion is permanently blunted if CD4^+^ T cells or B cells are transiently impaired at the time of transduction in AAV-HBV mice, indicating that HBV persistence and high antigen environments impair functional immune responses. Thus, therapeutic vaccine design should consider CD4^+^ T cells as a target to overcome immune tolerance and viral persistence.

Indispensable to T cell help is signal transduction through the CD40/CD40L axis. The ligation of membrane-bound CD40 on B cells induces downstream signaling cascades that upregulate pro-survival and pro-maturation transcriptional programs and support germinal center formation (28, 41). Blocking the CD40/CD40L axis disrupts HBsAg clearance and HBsAb production, likely due to reduced HBsAg-specific IgG-producing B cells. Unexpectedly, CD40L blockade also appears to mitigate HBV gene expression determined by reduced peripheral HBeAg compared to control mice. This effect is recapitulated in CD4-depleted mice and is unlikely to result from a humoral response against HBeAg, as HBV mRNA expression is similarly reduced (data not shown). These observations suggest interactions involving CD4^+^ T cells through the CD40/CD40L axis that may regulate HBV replication beyond humoral immunity.

CD40 expression is not limited to B cells but is also found on professional antigen-presenting cells (APCs) such as dendritic cells and macrophages (27). The function of CD40 on APCs is similar to B cells in that ligation by CD40L induces pro-survival and pro-maturation programs. Functionally, this process, known as DC licensing, permits maturation and cross-presentation to support antiviral cytokines and effector CD8^+^ T cell responses (42, 43). Previous studies using HBV transgenic mice revealed HBV control following CD40 activation through cytokine-mediated and T cell-independent mechanisms (44). CD40 activation by agonistic mouse antibody FGK4.5 has been shown to induce functional, antigen-specific immune responses in tumor models and viral infections (45 – 48), supporting the possibility of using this approach to treat HBV. We are now using AAV-HBV mice to further investigate whether CD40 signaling can be exploited to induce CD8-dependent HBV control.

## MATERIALS AND METHODS

### Mice

BALB/c (stock #000651) mice were purchased from The Jackson Laboratory. Six- to eight-week-old male mice were used for AAV-HBV studies. Mice were housed in the Animal Resource Facility at Albany Medical College. All experiments followed protocols approved by the Albany Medical College Institutional Animal Care and Use Committee.

### *In vivo* antibody administration

Depleting [α-CD8β (53–5.8); α-CD20 (MB20-11); α-CD4 (GK1.5)], blocking [α-MHCII (M5/114); α-FcγR CD16/CD32 (2.4G2)], or control [α-keyhole limpet hemocyanin (LTF-2)] antibodies from BioXCell were diluted in 1 × PBS and administered twice weekly beginning 1 week before AAV-HBV transduction for a total of 2 or 10 doses (225 µg/dose).

### AAV-HBV transduction

AAV serotype 8 encoding a 1.2-mer HBV genome (Genotype D) was prepared by SignaGen. HBV replication was initiated in male BALB/c mice by transduction via retro-orbital injection of 4–5 × 10^10^ genome copies of AAV-HBV diluted in 200 µL 1 × PBS to generate peak serum HBsAg levels of approximately 2,000 ng/mL.

### Immunization strategy and adoptive transfer

Seven- to ten-week-old BALB/c mice were used for immunization and adoptive transfer of splenocytes. Mice used for immunization were DNA primed with 50 µg of middle hepatitis B surface antigen (MHBs)-expressing plasmid and boosted 3 weeks later with 1 × 10^6^ PFU of recombinant vesicular stomatitis virus (VSV) encoding MHBs (VSV-MHBs) (49). Splenocytes from immunized or naïve mice were harvested 2 weeks following the VSV-MHBs boost. Briefly, spleens were mechanically dissociated and underwent RBC lysis for 5 min at room temperature. Splenocytes were washed and resuspended in 1 × PBS before enumeration. 5 × 10^7^ naïve or immunized live splenocytes were transferred into recipient mice 11 weeks post-transduction.

For CD4^+^ T cell and B cell isolation, EasySep mouse CD4^+^ T cell and EasySep mouse B cell isolation kits (STEMCELL Technologies) were used according to the manufacturer’s recommendations. Briefly, 2 × 10^8^ splenocytes from immunized mice were subject to magnetic separation. Multiple rounds of purification were pooled together to generate a total of 6 × 10^7^ B cells and 3.4 × 10^7^ CD4^+^ T cells. Recipient mice received either 7.5 × 10^6^ B cells or 4.25 × 10^6^ CD4^+^ T cells in a 200-µL retroorbital injection 9 weeks post AAV-HBV transduction. Cell fraction purity was determined by flow cytometry.

### B cell ELISPOT

Mouse IgG/IgM and IgG1/IgG2a Double-Color ELISPOT Assays (Cellular Technology Ltd.) were used following the manufacturer’s recommendations. Briefly, plates were coated with 1 µg/well HBsAg (Fitzgerald Industries) or 1 µg/well Bovine Serum Albumin (BSA) overnight before adding 2 × 10^5^ unstimulated splenocytes per well. Spot-forming cells (SFC) were enumerated using an automated spot counter (Cellular Technology Ltd).

### ELISA

Serum HBsAg, HBeAg, HBsAb, and HBcAb were measured by ELISA (International Immunodiagnostics) following the manufacturer’s protocol. Recombinant HBsAg (subtype ayw) and HBeAg protein standards were purchased from Fitzgerald Industries.

### Liver HBV gene expression (RT-qPCR)

Snap-frozen liver samples from the designated experimental endpoint were homogenized in RLT buffer supplemented with 2-mercaptoethanol, and RNA was purified from liver homogenates using an RNeasy mini kit (Qiagen) according to manufacturer recommendations. Reverse transcription was performed in 20 µL reactions using a High-Capacity cDNA Reverse Transcription Kit (ThermoFisher) according to the manufacturer’s recommendations. TaqMan Fast Advanced Master Mix (ThermoFisher) was used for quantitative PCR. Reactions were done on a QuantStudio 6 real-time PCR system (ThermoFisher) using QuantStudio Design and Analysis software v2. The following primer sequences were used: HBV probe 5′-CCT CTT CAT CCT GCT GCT ATG CCT CAT C-3′, antisense 5′-GAC AAA CGG GCA ACA TAC CTT-3′, sense 5′- GTG TCT GCG GCG TTT TAT CA-3′ (50). HBV RNA expression was normalized to GAPDH (ThermoFisher, Assay Mm99999915_g1).

### HBV DNA qPCR

HBV DNA was processed from serum using a High Pure Viral Nucleic Acid Kit (Roche) according to the manufacturer’s recommendations. Briefly, 20 µL of serum diluted in 1 × PBS and supplemented with proteinase K was incubated at 72°C for 30 min. Samples were then processed with spin columns, and purified DNA was eluted with water. qPCR for HBV was performed using a plasmid encoding the HBV genome to generate a standard curve. TaqMan Fast Advanced Master Mix (ThermoFisher) was used for HBV quantitative PCR reactions, as described above.

### Flow cytometry analysis

Spleen, liver, and blood were utilized for cell quantification and phenotyping. Blood was initially collected in 10 mM EDTA in PBS to reduce clotting. Following euthanasia, the spleen or liver was collected in RPMI medium containing 1% fetal bovine serum (FBS). Tissues were homogenized by pressing through a 70-µM cell strainer. Cells were washed in RPMI containing 1% FBS and resuspended in ACK lysing buffer for RBC lysis. Leukocytes were isolated from perfused liver by resuspending liver homogenates in 40% Percoll and centrifuging at 560 × *g* for 15 min. Cells were washed and resuspended in 50 µL FACS buffer and stained with antibody (diluted 1:100). CD4^+^ T cell phenotyping was performed using a mouse T_H_1/T_H_2/T_H_17 phenotyping kit (BD Pharmingen) according to the manufacturer’s recommendations. Other antibodies for flow cytometry were purchased from Biolegend (α-CD3, CD4, CD69, CXCR5, PD-1), BD Biosciences (α-CD19), and ThermoFisher (α-CD8, CD19). Briefly, purified leukocyte cell suspensions from the spleen and liver were stimulated with PMA (50 ng/mL) and ionomycin (1 µg/mL) in the presence of BD GolgiStop (monensin, 4 µL/6 mL) for 5 h at 37°C. Cells were washed and resuspended in 50 µL FACS staining buffer before adding 20 µL antibody cocktail and allowed to incubate for 30 min at room temperature. Sample acquisition occurred using a FACSymphony A3 cytometer (BD), and final analyses were completed using FlowJo software (version 10.7).
